# ‘It doesn’t happen how you think, it is very complex!’ Reconciling stakeholder priorities, evidence, and processes for zoonoses prioritisation in India

**DOI:** 10.3389/fpubh.2023.1228950

**Published:** 2023-08-22

**Authors:** Festus A. Asaaga, Aditi Sriram, Mudassar M. Chanda, Subhash L. Hoti, Juliette C. Young, Bethan V. Purse

**Affiliations:** ^1^UK Centre for Ecology & Hydrology, Maclean Building, Wallingford, United Kingdom; ^2^One Health Trust, Obeya Pulse, Bengaluru, Karnataka, India; ^3^ICAR-National Institute of Veterinary Epidemiology and Disease Informatics, Bengaluru, Karnataka, India; ^4^ICMR-Vector Control Research Centre, Pondicherry, India; ^5^Agroécologie, INRAE, Institut Agro, Univ. Bourgogne, Univ. Bourgogne Franche-Comté Dijon, Dijon, France

**Keywords:** zoonosis, disease prioritisation, health policy, India, low- and middle-income countries

## Abstract

**Background:**

Why do some zoonotic diseases receive priority from health policy decision-makers and planners whereas others receive little attention? By leveraging Shiffman and Smith’s political prioritisation framework, our paper advances a political economy of disease prioritisation focusing on four key components: the strength of the actors involved in the prioritisation, the power of the ideas they use to portray the issue, the political contexts in which they operate, and the characteristics of the issue itself (e.g., overall burdens, severity, cost-effective interventions). These components afford a nuanced characterisation of how zoonotic diseases are prioritised for intervention and highlight the associated knowledge gaps affecting prioritisation outcomes. We apply this framework to the case of zoonoses management in India, specifically to identify the factors that shape disease prioritisation decision-making and outcomes.

**Methods:**

We conducted 26 semi-structured interviews with national, state and district level health policymakers, disease managers and technical experts involved in disease surveillance and control in India.

**Results:**

Our results show pluralistic interpretation of risks, exemplified by a disconnect between state and district level actors on priority diseases. The main factors identified as shaping prioritisation outcomes were related to the nature of the zoonoses problem (the complexity of the zoonotic disease, insufficient awareness and lack of evidence on disease burdens and impacts) as well as political, social, cultural and institutional environments (isolated departmental priorities, limited institutional authority, opaque funding mechanisms), and challenges in organisation leadership for cross-sectoral engagement.

**Conclusion:**

The findings highlight a compartmentalised regulatory system for zoonoses where political, social, cultural, and media factors can influence disease management and prioritisation. A major policy window is the institutionalisation of One Health to increase the political priority for strengthening cross-sectoral engagement to address several challenges, including the creation of effective institutions to reconcile stakeholder priorities and prioritisation processes.

## 1. Introduction

Global health security concerns surrounding threats from endemic and emerging zoonotic diseases – that cycle between animals and humans – are high on international and national policy agendas. Expanding human populations and sustained land-use change have brought humans into close proximity to wildlife, amplifying the risk of potential disease spillover from wildlife to livestock and humans (1, 2). At the same time, there is the resurgence of endemic zoonotic infections in new geographical areas, often with disproportionate health, social and livelihood impacts on poor and vulnerable groups in low-and middle-income countries (LMICs) that have high dependence on ecosystems (3–6). Available statistics suggest that the direct cost of zoonotic diseases between 2000 and 2010 (prior to the coronavirus disease (COVID-19) pandemic) exceeded $20 billion with over $200 billion indirect losses to affected economies globally (7). As zoonoses often transcend geographical and political boundaries requiring coordinated responses (8), there has been a call to strengthen cross-sector engagement to better prioritise and target interventions, particularly in many LMICs saddled with poor surveillance programmes and limited institutional capacity (9–11).

Against this background, defining disease priorities for resource allocation and intervention is complex and deeply political (12–14) and also bureaucratic, requiring more than just expert assessment. In thinking strategically about priorities, it is important to consider the way the prioritisation processes are being shaped by the political and institutional contexts, and what this means for the intended outcomes and planned interventions. It is equally important to address fundamental questions such as: (i) which objectives should disease prioritisation seek to achieve, (ii) which candidate diseases should be prioritised, (iii) who should be involved in prioritisation, (iv) how should decision-makers prioritise, (v) what are the evidences for prioritization, and (vi) what priorities should be assigned?

Addressing questions like these requires an integrated approach that provides the analytical lens or platform to reconcile different stakeholder priorities, available evidence and institutional processes that shape prioritisation outcomes. Recent prioritisation efforts have focussed on bridging the gap between animal and human health sectors through the agency of collaborative, cross-sectoral expert-driven prioritisations (5, 15). Although such cross-sectoral expert-driven approaches (partly motivated by evidence gaps on disease burdens and multi-sectoral impacts) have proved useful (5, 15); there is often an over-emphasis on the technical aspects of disease prioritisation overlooking the intricate political economy factors that shape outcomes of such expert-driven prioritisations (13, 16). This has meant that disease prioritisation outcomes often fail to generate a holistic picture of the burdens and scale of multi-factorial social, economic and environmental impacts, necessary to inform the effective contextualisation of disease interventions (13, 15, 17). In addition, authors have argued that solely expert-driven disease prioritisation often privilege known exotic and epidemic diseases at the expense of endemic high burden neglected diseases with far-reaching negative ramifications for livelihoods and local health systems (9, 18).

In the face of limited resources for disease prevention activities (e.g., biosecurity, vaccination, chemotherapeutics, public education, and surveillance) and control measures, a better understanding of the political, institutional, and socioeconomic factors that affect prioritisation decision-making and outcomes across different contexts remains paramount in guiding defensible resource allocation and optimising interventions. While some previous studies have identified factors that shape political prioritisation and responses to non-communicable diseases (12, 16), to our knowledge, there has yet to be a systematic exploration of the contextual factors that have shaped political priorities for zoonoses and opportunities for cross-sectoral disease prioritisation decision-making in LMIC settings. As Buse et al. (19) observe, health policies are products of complex inter-relationship of context, process and actors. Drawing inspiration from Lemmi (16) and Heller et al. (12), we applied Shiffman and Smith’s (20) framework on determinants of political priority for global health issues to explore the dynamics of zoonotic disease prioritisation for optimising resource allocation and interventions in India. We ask the overarching question: *why do some zoonotic diseases receive priority from policy decision-makers and health planners (responsible for disease surveillance and control) whereas others receive little attention?* Specifically, we address the following sub-questions: (i) how are zoonotic diseases prioritised for interventions and (ii) what are the knowledge gaps affecting disease prioritisation?

## 2. Materials and methods

We conducted a qualitative study using semi-structured interviews with policy decision-makers, disease managers and technical experts involved in zoonoses management in India across national, state and district levels. The selection of key-informants was based on a prior One Health stakeholder mapping exercise conducted to identify different organisations involved in disease surveillance and control. Stakeholders in this context refer to individuals or actors whose interest (or mandate) significantly affect or are impinged by management decisions regarding zoonoses prevention and control. We purposively selected key-informants to cover the primary roles in the policy agenda setting and implementation of interventions across scale. Other actors and institutions were recruited through snowballing. Recruitment of key-informants for the in-depth interviews continued until data saturation was reached, stratified by sector (animal and human health, forestry and wildlife sectors) to capture population heterogeneity (9, 16). We carried out 9 key-informant interviews before the COVID-19 outbreak in India (in early 2020) and a further 17 interviews between December 2021 and May 2022. Interviews were face-to-face and by zoom (depending on participant’s preference) and lasted an average of 45 min. Informed consent was sought from participants in writing or orally before the interviews. In instances where participants gave consent verbally, these were duly recorded in a study ethics inventory for record keeping. Interviews followed a discussion guide that comprised four thematic areas that were core to the study: background of key-informants, prioritisation of zoonoses in policy and knowledge gaps, decision-support tools for zoonoses management, and cross-sectoral collaboration for zoonoses management.

We adopted Shiffman and Smith’s political prioritisation framework to organise interview data into themes for evaluating factors shaping the political prioritisation of zoonoses in India (20). The modified framework includes 4 themes and 11 sub-themes: actor power (policy community cohesion, leadership, guiding institutions and civil society mobilisation), ideas (internal frame and external frame), political contexts (policy windows and disease governance structure), and issue characteristics (credible indicators, severity and effective interventions) (Table 1). All interviews were digitally recorded and transcribed verbatim followed by anonymization using pseudonyms to safeguard participants’ confidentiality (see Table 2 and Supplementary Table 1; Supplementary Figure 1).

**Table 1:** Modified framework on determinants of political priority for zoonoses based on Shiffman and Smith (20).

	Description	Factors shaping policy prioritisation
Actor power	The strength of the individuals and organisations concerned with zoonoses management	Policy community cohesion: the degree of collaboration/ unity between various actors that are centrally involved in zoonoses management across scale. Leadership: the presence of actors or individuals capable of fostering cross-sectoral collaboration and acknowledged as particularly strong champions for the cause. Guiding institutions: the effectiveness of organisations or coordinating mechanisms with a mandate to lead cross-sector prioritisation process. Civil society mobilisation: the extent to which non-government organisations and media have mobilised to press international and national political authorities to focus attention on the disease at the national and state levels.
Ideas	The ways in which those involved with zoonoses management understand and portray the problem	Internal frame: the degree to which designated actors agree on the definition of, causes and solutions to the disease problem External frame: public and media portrayals of the disease problem that resonate with external audiences, especially political leaders who control resources
Political contexts	The environments in which actors operate (in terms of formal or informal rules and norms that shape actions)	Policy windows: political moments when global or national conditions align favourably for disease problem(s), presenting opportunities for advocates to influence decision-makers Disease governance structure: the degree to which norms and institutions operating in a sector provide a platform or incentivises decision-makers
Disease characteristics	Features or attributes of the disease problem	Credible indicators: clear measures that show the overall burden of the disease and that case be used to monitor progress Severity: the size of the burden relative to other disease problems, as indicated by objective measures such as incidence, mortality levels, case fatality rates Effective interventions: the extent to which proposed means of tackling the disease are evidence-based, cost effective, available, simple to implement, and easily understood by decision-makers

**Table 2:** List of stakeholders interviewed by sector.

Interviewee domain	Type of power/influence	Influence on prioritisation	Number of informants	Experience level	Pseudonyms
Policy-Central government (Animal health)	Decision-makers and influence policy formulation	High	1	26 years	Central-HH
Policy-Central government (Human health)	Decision-makers and influence policy formulation	High	2	15–25 years	Central-AH
Policy-State government (Animal health)	Decision-makers and influence policy formulation/implementation	High	2	22–25 years	State-AH
Policy-State government (Human health)	Decision-makers and influence policy formulation/implementation	High	5	20–35 years	State-HH
Policy-State government (Wildlife)	Decision-makers and influence policy formulation/implementation	High	1	11 years	State-W
Implementation-District government (Human health)	Decision-makers and influence policy implementation	Low-moderate	7	3–15 years	District-HH
Implementation-District government (Forestry)	Decision-makers and influence policy implementation	Low-moderate	1	10 years	District-F
Implementation-District government (Wildlife)	Decision-makers and influence policy implementation	Low-moderate	1	15 years	District-W
Academic or Research	Knowledge generation and influences policy formulation	Low-moderate	5	20–30 years	Academia
Non-governmental representatives	Practitioner and influences policy implementation	Low-moderate	1	12 years	NGO

The anonymised interview transcripts were analysed thematically within NVIVO 12 using a combination of deductive and inductive analysis, with the individual as the unit of analysis (21). We first coded the interview data based on the three thematic areas based on the interview guide [(i) who is involved in the governance system related to zoonotic disease prioritisation, (ii) how are zoonotic diseases prioritised, (iii) what are the key knowledge gaps affecting the disease prioritisation process?]. The research team developed a coding framework based on initial codes identified from five randomly selected transcripts. After the pilot coding exercise, two authors (FA and AS) double-coded the rest of the interviews and disagreements were resolved through team discussions. We used the Shiffman and Smith (20) framework meta-themes for the final analysis and data interpretations. To better capture the original ‘perspectives or voices’ of zoonoses actors and decision-makers themselves on the state of play for zoonoses prioritisation, we draw heavily on verbatim illustrative quotations in the presentation of the study findings. Ethical approvals were obtained from the Research Ethics Committees of the Ashoka Trust for Research in Ecology and the Environment (IRB/CBC/0003/ATV/07/2018), the Institute of Public Health (IPH) Bangalore (IEC-FR/04/2017) in India, and received a favourable ethical opinion from the Liverpool School of Tropical Medicine Research Ethics Committee (reference numbers 17-062 and 20-051) in the United Kingdom.

## 3. Results

### 3.1. Participant demographics

In total, 26 interviews were conducted and categorised into four sectoral domains. The majority of informants were male and had over a decade of experience working in the field of zoonoses. Despite efforts to achieve a balanced representation of relevant sectors in zoonoses decision-making across levels, over half of informants (53.8%, *n* = 14) were affiliated to the human health sector, indicative of the dominance of human health actors in India’s health policy arena. Three informants were affiliated with the animal health and two with the wildlife sectors, respectively. The remaining six were experts and other informed actors, with extensive knowledge having spent their careers working on zoonoses in academic institutions (e.g., researchers) and civil society organisations focussed on disease interventions. Table 2 summarises the sectoral affiliation, jurisdiction and level of policy influence of the study informants. The findings from the interviews are presented thematically according to the Shiffman and Smith (20) political prioritisation framework with illustrative quotations in the ensuing paragraphs.

### 3.2. Zoonoses prioritisation and decision-making in India

Reflecting India’s federated system of governance, different levels of government regulatory agencies and bodies are responsible for zoonoses management, exemplifying a complex and compartmentalised institutional architecture for disease prevention and control (9). Although health is a state subject, the cross-sectoral coverage of zoonoses has meant that different layers of state and national-level authorities are involved in the prioritisation decision-making process. From the interviews, informants concurred that aside from broad World Health Organisation (WHO) guidelines and the Integrated Disease Surveillance Programme (IDSP) report informing the prioritisation process (14), there are no nationally ratified disease prioritisation criteria. Respective state governments have the legal mandate to determine their own priorities based on local needs, capacity and context. Two typical views, respectively, expressed by a national and state-level bureaucrat on the disease prioritisation process are illustrative:

*“Officially we do not have such a ranking system which is top, what is next; we do not have a ranking system. If, for example, dengue cases are more then it will come to the top and this is informal …”* (KI002, Central-HH).

*“We are able to prioritise ourselves as per the local need but common diseases are prioritised at national level. Local diseases are prioritised at local level. That liberty is given to the states. Some states have added diphtheria, others have added pertussis. Karnataka, for example, has added Kyasanur forest disease (KFD) as a priority disease.”* (KI005, State-HH).

On the rationale for the state-centric prioritisation of diseases, another state-level informant explained that this was mainly to avert any potential dilution effect (often associated with the operationalisation of national policies at the local level) and to foster strong state ownership of the process:

*“If it [prioritisation] is done at the national level, then dilution will be there. Suppose there are 5 diseases, I will make a plan for 5 diseases. My priority, now at present because of KFD outbreak and H1N1, my top priority is H1N1 and second is KFD. So I have to prioritise my action plan for the control of morbidity and mortality of these two diseases. So better to give at the state level …”* (KI006, State-HH).

### 3.3. Actor power

To the extent that guiding institutions, actor relationships and power shapes the disease system and prioritisation outcomes, it was important to assess the strength/authority and positionality of actors and organisations within the policy arenas responsible for zoonoses agenda-setting and interventions. From the interview data, three types of authority – institutional, delegated and expert authority – were identified as linked to zoonoses management (22). The analysis revealed that there were differences in the way these authorities were asserted by government, technical experts and civil society actors in zoonoses decision-making. In this regard, authority assertion was manifested as claimed authority (assertions by actors on a particular type of authority), circumscribed authority (assertions of limited or restricted authority), and/or contested authority (actors disputation or perception of other actors’ authority) (23).

The key actors within the health system identified as wielding institutional authority regarding zoonoses control and prevention include the National Centre for Disease Control (NCDC), Indian Council for Medical Research (ICMR), Indian Council for Agricultural Research (ICAR), Department of Animal Husbandry and Dairying (AH&D) and state departments of animal, human health and wildlife (9). State-level informants particularly perceived the NCDC as claiming circumscribed institutional authority in that its role is limited to technical oversight and support especially during disease emergencies. At the sub-state level, disease managers exercised delegated authority in terms of outbreak response and other intervention-related activities. Expert authority was mainly exerted through the provision of scientific evidence on focal diseases and expertise on criteria used to rank candidate diseases for intervention. Actors that claimed expert authority include federal and state-level government departments of health, technical experts and civil society.

Concerning the degree of collaboration between various actors involved in zoonoses prioritisation, national and state-level informants generally concurred that the disease prioritisation process is largely sectorally-driven, i.e., the emphasis of a focal zoonosis that is considered of prime importance for animal health may not be the same for human health. On the rationale for the siloed-approach to prioritisation decision-making, a national-level animal health actor said:

*“Glanders, for example, is a category B bioweapon which from my perspective is a priority disease. Okay you go to the human health side, the rate of transmission to human is very less. Now, how do you prioritise from that side?”* (KI001, Central-AH).

On the question over what opportunities exist for joint disease prioritisation, the interviews revealed mixed views as to the extent to which decision-making process was indeed collaborative. For instance, animal health actors particularly highlighted that existing collaborations were largely tokenistic as they often played second fiddle to their human health counterparts with respect to zoonoses prioritisation. Two critical views expressed by a state-level actor and an expert from the animal health sector about collaborative prioritisation in the current state of affairs are instructive:

*“They [human health actors] will not be at all ready to listen to you. If you go to them and you tell them this is what we need to do, they will not at all be willing to listen to you. Actually the policy matters have been directed towards protection of mankind. We are working to protect humankind, aren’t we [sic]? And the animal health side is completely side-lined.* (KI008, State-AH).

*“There is missing link between human health and animal health sectors [in respect to collaborative decision-making]. They will not take our ideas and we will not take theirs. It is recently only we came together …”* (KI004, State-AH).

From the human health perspective, some state-level informants concurred that their animal health counterparts had circumscribed authority with respect to zoonoses decision-making since the supposedly joint-prioritisation platform was largely human health centred. Interviewees contended that the power asymmetries shaping collaborative arrangements between animal and human health actors are reflective of a broader social stratification, in which human doctors exerted high societal influence relative to veterinarians (9). Besides, other informants reflected that although technical staff want to work collaboratively given the cross-sectoral nature of zoonosis problems, disparate departmental priorities, overstretched staff and funding modalities have meant that joint prioritisation of diseases for intervention is often challenging. One state-level bureaucrat disclosed, “*every sector remains overloaded. For example, if I talk to colleagues in the veterinary sector, I know that human resources is lesser than what is required. So that is a limiting factor. So people remain bounded with their own departmental work that they do not have time, energy and resources to engage other sectors, except during disease emergencies. It may not necessarily be only in India”* (KI009, State-HH). Nevertheless, other informants downplayed the contested authority of animal health actors in joint prioritisation commonly citing examples of successful cross-sectoral engagement (e.g., data-sharing between departments) and sustained informal relationships which contributed significantly in circumventing otherwise ‘formal barriers’ to collaborations (KI002, Central-HH).

Regarding the interplay between civil society mobilisation and prioritisation outcomes, several national and state-level interviewees highlighted that advocacy work elevated the position of certain candidate diseases and certain drivers influenced their visibility. Citing the example of rabies, national-level actors reflected on how the celebration of World Rabies Day at the national level had shaped institutional consciousness about rabies and its political visibility relative to other reported zoonoses. Nevertheless, civil-society engagements around neglected diseases particularly remain weak, hence their low priority/visibility in the policy context (KII013, NGO). As one bureaucrat lamented, “*nobody is talking about these neglected disease (say KFD or scrub typhus) and there is no funding to incentivise related interventions. Unlike rabies or TB, national health control and civil society engagement has been weak for other diseases*” (KII006, State-HH). Correspondingly, a national-level bureaucrat (from the animal health side) recounted how commercial livestock and dairy farms constituted a strong lobbying group that influenced the political prioritisation of brucellosis for eradication by 2030, culminating in the announcement of funding for the National Animal Disease Control Programme (NADCP) by the Prime minister’s office for 2019–2024 (24). The foregoing underscores the view that the extent to which a disease is prioritised depends on who has an interest in it. Rationalising the lack of an integrated policy for rabies (encompassing vaccination and dog management/elimination) for example, an expert informant recounted the significant role an influential animal welfare lobby played in shaping the rabies prioritisation policy:

*“I think because of the fact that the animal welfare lobby has usurped the agenda for dog population control. It is really to do with the animal welfare lobby … That’s why there has been no strong move to dog control by the health department to come up with any policy even though it is their mandate to reduce the burden of disease”* (KI011, Academia).

### 3.4. Ideas

Underpinning the disease prioritisation context is the resonating set of ideas – i.e. framing of the issue publicly in ways that attracts political support – that determine the attention accorded to the candidate priority disease. In essence, how a disease is portrayed and understood by actors involved in zoonoses management matters for prioritisation and intervention (20). We thus asked participants their perceptions about the framing used to characterise zoonoses and the interplay with prioritisation criteria and implementation of cross-sectoral interventions. From the interview analysis, it emerged that the portrayal of candidate zoonoses for prioritisation revolved around two frames – internal (i.e., the level of consensus with the policy community) and external (which is the public portrayal of the issue).

#### 3.4.1. Internal frame

A shared understanding of an issue and its solutions by a policy community is fundamental to its coalescence. Despite the lack of formally ratified disease prioritisation criteria, several interviewees highlighted morbidity, case fatality, mortality and economic impact as the common ‘internal framing’ used to characterise candidate diseases for prioritisation. Two typical observations by a state and national level official regarding the determinants of priority accorded to focal diseases are instructive:

*“The most prominent diseases are linked to high case fatality and mortality which informs their high priority status. Everywhere [in the country] importance will come to a [candidate] disease when any death happen from it, mortality is the primary criteria. Until then we do not take it seriously. Once the deaths happen State, national, international, and locally everyone will be alert*” (KI-005, State-HH).

*“I guess in most part of the community it is very pathogen-focused but there are a lot of similarities between different kinds of zoonotic diseases. Obviously the kind of visibility they get in the policy agenda and the kind of responses depends on who gets affected in terms of population, types of animals, economic or political visibility and also how visible the diseases are.”* (KI002, Central-HH).

#### 3.4.2. External frame

On the question of external framing of candidate priority diseases, a recurring theme across the interviews was that public attention and media publicity featured prominently in determining the political visibility or importance accorded to respective disease problems. Juxtaposing the case of two priority zoonoses (Anthrax and Brucellosis) in elucidating how public perception affects prioritisation outcomes, an expert informant and disease manager, respectively, said:

“*Anthrax causing deaths causes human outcry whereas you know Brucellosis might not in the same way for example, despite having a much larger burden. Rabies having smaller cases is much more visible compared to other silent diseases with larger burden”* (KI015, Academia).

*“If there is a huge number of animal deaths happening in the short span of time, for example the case of an anthrax outbreak people are bound to get incensed they are bound to inform their leadership so chances are that it will be high on the political ladder. But if a disease which is not causing huge deaths or is causing silent disability for a long time then people might or might not be aware about it even if the scientists are aware people might not be aware about it but it is a political issue”* (KI017, District-HH).

A number of national and state level actors (responsible for disease surveillance and control) also corroborated the foregoing observations, implicitly suggesting that prioritisation of diseases is not necessarily based on epidemiological characteristics of diseases (e.g., burden, outbreak potential, spread and impact) *per se* but largely the political visibility shaped by narratives about diseases and their portrayal in the media space. In separate interviews, a disease manager and surveillance officer addressed the effect of public portrayals or disease narratives on eventual priority setting outcomes:

“*Right now, the dengue positivity [for example] is more compared to that of COVID-19, but people are not much bothered about dengue. Also the health department is not giving that much importance, what COVID-19 is getting. The government prioritisation shifted based on how much news that disease is making or how much fear that is made in people’s minds*” (KI022, District-HH).

*“Most of the things are driven by the press also. Some press persons will magnify few diseases which are not so important. If highlighted in the media, once the world comes to know that is a very serious, these kind of people are really getting troubled then it is highlighted [within policy circles]. But unfortunately, the effort [political visibility] is sustained only initially that euphoria will be there …”* (KI028, District-HH).

### 3.5. Political contexts

#### 3.5.1. Disease governance structure

A general consensus across the interviews was the critical role the political environment – that is the overall institutional and cultural context in which actors operate (20) – plays in shaping actor priorities, decision-making process and outcomes. As relevant sectoral agencies for zoonoses management come under the jurisdictional scope of state governments, this has meant that zoonoses decision-making has been subject to the local politics of the state. Citing Tamil Nadu as a model for public health interventions to other Indian states, an expert informant argued that in states where health is high on the policy agenda and/or has a history of disease outbreaks or health emergencies, the bureaucratic machinery as well as the general public is sensitive to zoonotic disease-related concerns. Corroborating this observation, a state-level bureaucrat admitted that disease prioritisation was a function of internal politics as the positionality of bureaucrats renders them convenient tools for advancing political objectives of ruling regimes, thereby affecting decision-making processes. Expressing frustration about the somewhat lack of prioritisation of technical advice in critical policy deliberations, a surveillance officer remarked *“so obviously you know the setup is bureaucratic … most senior people heading relevant committees are not the technical people [but political actors] so other members are going to defer to that person’s you know politics. So there’s only so much you can achieve in an environment like that …”* (KII017, District-HH).

Another informant had this to say:

“*Whatever the activities that are implemented, the decision-maker should be a technical person. Not the bureaucrats as far as my knowledge is concerned … That is how we eradicated smallpox, polio and guinea worm because the technical persons were the heads of department in those days. That is the need of the hour!*” (KI009, State-HH).

A cross-section of interviewees also conceded that disease under-reporting or misreporting as a function of wider state-level politics significantly eroded the credibility of the existing prioritisation process. A state-level bureaucrat, in defence of the seemingly heavy political influence in disease prioritisation decision-making, argued that the notion of controlling the narrative was equally important as a disease itself. The said official further noted that the quest to safeguard the country’s reputation, avert unsolicited surveillance, economic losses and public outcry (as in the case of the 1994 plague outbreak) largely incentivised decision-makers’ attempts to control narratives by suppressing news of disease outbreaks. Relatedly, a national bureaucrat voiced out, “*they are not actually coming up with the truth! Some of the states are absolutely silent and saying like we do not have this disease although our informal surveillance networks suggest otherwise*” (KI002, Central-AH). In juxtaposition, several state and district level informants disclosed some implicit assumptions that shaped the existing disease reporting regime as some interviewees cited fear of victimisation/scapegoating, punitive-transfers and/or overstretched technical manpower as some probable reasons for the supposed reticence in full disclosure of ‘actual’ disease outbreaks to higher-ups at the national and state levels. In fact, some informants particularly observed that the reticence/behaviour of under-reporting has consequences for degree to which they can rely on existing disease reporting systems like the IDSP and/or Integrated Health Information Portal (IHIP) for early warning and targeting interventions, understanding factors driving disease emergence, as well as for overall disease prioritisation. A typical perspective by a state-level surveillance officer is illustrative:

“*In outbreak situations, it is the state or district level officers whose necks are on the line if they are not able to [in the eyes of political actors] properly control the situation. Technical experts sitting at national level institutions like National Centre for Disease Control [NCDC], you know, they will come and do outbreak investigations but if something happens they really do not have to face that level of accountability because their job is to provide technical advice and the decision-making and the political fallout of that is going to be restricted to state level officers.”* (KI007, State-HH).

Reflecting on the current state of play, some state and district level informants believed technical officers in some government departments and ministries (responsible for zoonoses decision-making) become ‘pawns’ often providing seeming scientific justifications for otherwise political decisions (25). A case in point is the decision to withdraw the administration of the KFD vaccine in all endemic regions as of October 2022, ostensibly to quell public outcry following widespread media reports of the vaccine’s shortcomings (26). Inferably, the seeming ‘politically motivated’ action somewhat overrides management efforts of disease managers who had earlier resorted to using an ‘unlicensed’ vaccine as the best available option to avoid severe clinical signs and death in affected populations in their jurisdictions, largely informed by epidemiological evidence of some effectiveness of the said vaccine in doing so (27, 28). Acknowledging the potential ramifications of lack of management option for KFD due to political pressure generated by the increased awareness of the existing vaccine shortcomings, a disease manager implicitly opined:

*“Managing media and elected representatives that becomes a very big burden. In a way it is good but sometimes it is very detrimental [for disease management]. Their [political actors] pressure is always reflected in the eventual decisions we make in the area of our work …”* (KI010, District-HH).

Aside from the political contexts, the cultural environment (manifested by the deep-seated cultural/ religious sensitives) impacted significantly on disease prioritisation process and outcomes. For example, a number of interviewees noted, *inter alia*, that the high political visibility of brucellosis is largely a product of the religious and economic significance of the ‘cow’ in Indian society. This has meant that culling as an intervention is frowned upon in Indian political discourse and a silent policy of ‘non-identification of diseased cattle’ has been adopted, given the potential to trigger distress selling and stigmatisation. A typical view in this regard was given by a zoonoses expert:

“*The cow is a political icon in Indian political discourse so you cannot cull cows and if they are diseased and you cannot mark them as diseased as you end up spreading distress selling … So the policy of the government now from what I understand is not to identify individual animals but to do pooling of sampling at the farm-level and if they do identify individual animal they ask for quarantine measures and not for culling or not for selling because they do not want the farmer to be stigmatized or to resort in distress selling of the animal …*” (KI021, Academia).

#### 3.5.2. Policy windows

While discussing political moments that shifted policy and facilitated stronger engagement among key actors responsible for zoonoses decision-making, several informants (*n* = 17 key-informants, interviewed after the COVID-19 lockdowns in December 2021) were in agreement that the COVID-19 pandemic raised awareness about zoonoses and provided a good political window of opportunity for galvanising and sustaining cross-sectoral action. Indeed, contrasting the observations across interviews highlight a common refrain “*COVID-19 has given impetus to the need of the hour*” (KI009, State-HH), suggesting the importance of leveraging joint technical and surveillance capacities as an avenue to bridge the long-standing gap between animal health and human health actors and decision-makers with respect to zoonoses management. Reflecting on their pandemic experiences, several informants recounted for example how the spontaneous collaboration of functionally disparate ICAR and ICMR institutional laboratories proved instrumental in the turnaround times for the processing of human COVID-19 samples and surveillance for speedy decision-making and intervention. The observations of national human health and state-level veterinarian during separate interviews are instructive:

*“In the case of COVID-19, many animal health laboratories were involved in testing of human samples when suddenly the infection came to the country. At that time there was a lot of pressure on human health infrastructure available for testing of COVID-19 human samples. I think more than one million human samples were tested by veterinary laboratories. By ICAR institute itself, more than 5.5 million have been tested so far, particularly for human …”* (KI003, Central-HH).

*“Due to the [COVID-19] pandemic, the people [decision-makers] have become sensitized to the One Health agenda and they do give importance to One Health, but having an implementable One Health model with clear-cut guidelines and outcomes, uh still is difficult to develop and implement on the ground”* (KI004, State-AH).

Some informants were unenthused or sceptical about the so-called ‘COVID-19 opportunity’ arguing that the seemingly spontaneous cross-sectoral engagement of animal and human health actors was not surprising as it was reflective of the often reactive approach to cross-sector collaborations during outbreak situations. Indeed, to buttress their argument some local-level informants noted the missed opportunity in capitalising the learnings and experiences of the 2008 Avian Influenza outbreak management (widely touted as successful in terms of cross-sectoral action) to inform a concrete and holistic policy on cross-sectoral collaboration for improved zoonoses management. Other informants noted that the focus on COVID-19 response relegated otherwise equally urgent zoonoses problems to the background due to overstretched personnel and surveillance infrastructure. Two typical views in this regard by a disease expert and surveillance officer are illustrative:

*“I definitely feel that there is traction in understanding [about cross-sectoral action for zoonoses control] but again at the national level, the picture is much more complicated because there are so many different players and they are pushing so many different kinds of agendas that is difficult to make sense of.”* (KI015, Academia).

*“We were just about to take off in terms of Integrated Health Information Platform (IHIP) but when COVID-19 came, other diseases came into the background. So now that COVID-19 has come down to a tremendous extent, we want to bring this general disease surveillance to the forefront now”* (KI005, State-HH).

### 3.6. Characteristics of the zoonotic disease problem

Across the interviews, the biological, social, and economic characteristics of focal zoonoses were highlighted by participants as significant determinants of the degree of political visibility. Of the several disease-specific attributes identified, credible indicators (i.e., overall burden in focal areas in absolute terms), disease severity (i.e., relative *per capita* rate of infection, morbidity or mortality of candidate diseases) and effective interventions ranked highest across the interviews. Several interviewees cited the lack of credible indicators – clear measures that demonstrate the severity of candidate diseases impacted significantly on which candidate diseases gets prioritised – due to limited scientific evidence particularly on the multi-scale burdens, affected populations and socio-economic impacts of focal zoonoses. The foregoing observation further buttresses the earlier argument that politically induced reticence to record some problematic diseases within disease reporting systems and the state level prioritisation of which diseases are surveilled makes it difficult to judge overall burdens and compare data between states (see Section 3.5). As one interviewee, a state-level bureaucrat, openly admitted:

*“There are very sparse or few disease-specific studies because once we are ranking the diseases we require disease-specific data for that particular disease in different areas. As such [existing] prioritisation is currently not based on any solid evidence so it is difficult for people [disease managers] to follow it …”* (KI003, Central-HH).

Corroborating this assertion, other national and state level bureaucrats who were interviewed further clarified that the data quality rather than data availability *per se* constituted the key hurdle for prioritisation efforts as they often resorted to passive surveillance (e.g., disease incidence and outbreak occurrence in other areas) to inform decision-making rather than active, systematic surveillance mechanisms (as in the case of rabies, dengue and Japanese Encephalitis). Nuancing this narrative, some experts and state-level respondents further iterated that data challenge was largely due to the limited technical and surveillance infrastructural capacity at the district levels. Reflecting on how the limited surveillance capacity on disease reporting (particularly for the animal health perspective) affects data quality and prioritisation, an expert informant said:

*“We still lack data in terms of animal diseases under surveillance because it’s just the veterinarians who whatever they detect without much of a laboratory base, they just report it. On the human side, we do have infrastructure and we do have some manpower which I think still lacks some of the skilled manpower to actually do a decent reporting.”* (KI023, Academia).

A district level surveillance officer also argued that the limited technical capacity of field officers hampered their effective use of collated data. The said official observed:

“*Even if data is there, I am not able to make the right use of the data and I’m not analysing. Because there is a gap in the building capacity at the field functionary level as we have not inculcated the habit of analysing data to my field functionaries, so they never look at the data.”* (KI018, District-HH).

Corroborating observations about data limitations and quality, a top-level national bureaucrat (affiliated to the human health sector) admitted that while the IDSP has been useful for collating data on disease incidence and outbreak occurrence, a major drawback is the periodicity issues stemming from delayed/ and or non-submission of data by field functionaries:

*“The integrated disease surveillance programme [IDSP] has been very useful in terms of data collection, but there are some kind of you know periodicity issues with data as some reporting units are not submitting timely data. Besides, data picked by IDSP is not appropriate because most of the times, the diagnostic facility itself is not available. So we cannot at all be assured of the data quality.”* (KI003, Central-HH).

Yet some state-level informants, particularly from the health department did not perceive disease surveillance and data considerations to be a challenge. In fact, one interviewee, a senior state bureaucrat, in his explanation retorted *“not in Karnataka! No challenges like that. Our staff and medical officers are well aware of these diseases …”* (KI005, State-HH). The conflicting view of top-level bureaucrats and district-level implementers on data considerations highlights a potential disconnect between policymakers and implementers on the ground. The deep-seated sensitivities around open communication about challenges and need to safeguard their positions has meant senior-level bureaucrats often play to the “politics of the game.” Alluding to this assertion in separate interviews, a state-level surveillance officer and district-level disease manager, respectively, stated:

“*You have to realise that whenever we talk about zoonoses management [in India] is much easier to talk when you are not [directly] involved, it’s very complicated …”* (KI007, State-HH).

*“People who are sitting at the state/national level and who are capable of taking these decisions, sometimes they are also more burdened. At the same time, they do not really know the practical difficulties that we [disease managers] face at the primary health sector. If they get tuned to this kind of situation that’s there, I think they might agree with us. At the same time, district- level officers who are there they need to keep on escalating these questions to the people [decision-makers] who are there at the state level”* (KI012, District-HH).

On the question of how the degree of disease severity affected prioritisation outcomes, several informants suggested that the framing and measurement of severity is often quite ambiguous (due to complexity of zoonoses), but generally acknowledged that disease burdens, case fatality and mortality are critical determinants of political visibility of candidate diseases. As a district-level disease manager summarily noted, *“it’s mainly fatality! Otherwise even if a disease is causing any morbidity, it should cause a severe disability or mortality then only the district and state level actors start noticing”* (KI010, District-HH). Echoing similar sentiments, another expert informant argued: “*Bird flu, for example, comes like an epidemic whereas rabies and brucellosis are endemic (day-to-day problem). Whereas bird flu, it strikes and a lot of birds die, there is a lot of news in the newspaper, on the TV and the scare that it can spread to humans … In that case you know very fast action is to be taken by animal husbandry department so they also become very active*” (KI024, Academia). It therefore follows that existing prioritisation often tends to privilege exotic and more prominent diseases (albeit with smaller number of cases) at the expense of some high burden endemic diseases. Moreover, some local-level informants suggested that who a disease affects in terms of population and/or types of animals tended to significantly influence prioritisation outcomes. A case in point is KFD (an endemic viral haemorrhagic fever affecting forest-based communities in the Indian Western Ghats) which until 2019 had little visibility on the policy radar in Karnataka state. Citing the example of brucellosis, an expert informant observed different risk perception of the same disease by actors in the animal and human sectors leading to differential prioritisation:

*“I would say it depends on the pathogen and disease involved. The same disease might affect different people differently. For example, if a disease affects cattle (say brucellosis) then the dairy producers might have a different take (interested in productivity) compared to veterinarians who might perceive differently based on the risk of getting the disease. So I think there is a tension at play here between productivity and risks. Sometimes it is easier to tease them apart …”* (KI011, Academia).

The above observation might explain the seeming neglect of human anthrax outbreaks (despite its high prioritisation by Department of Animal Husbandry) often reported amongst marginalised tribal populations and workers handling animal carcasses (25, 29). Furthermore, the interviews highlighted the existence of effective interventions as vitally important in determining which diseases get prioritised. Several informants acknowledged that in light of competing diseases problems, the level of cost-effectiveness and ease of implementation of proposed interventions for a candidate disease influences its priority status. National and state level respondents, particularly from the animal health sector for example perceived rabies as a low hanging fruit given the perceived high potential for disease elimination and the high human and animal cost of inaction. This has meant that preventable zoonotic diseases (e.g., rabies) having smaller burdens are much more visible compared to under reported or under diagnosed diseases that currently lack effective forms of prevention (17).

## 4. Discussion

Understanding the political economy of factors that shape zoonoses prioritisation for resource allocation and intervention necessitates a contextual characterisation of the complex web of actors and networks. This includes their different constraints, priorities, mandates, and sources of information (12, 16, 17, 23, 30). This is predicated on the assumption that reconciling stakeholder priorities, evidence and processes is an essential prerequisite for effective prioritisation and management of zoonoses. While studies have examined the dynamics of cross-sectoral collaboration for zoonoses management (9, 15, 31), to our knowledge, how contextual and institutional factors interact to shape disease prioritisation outcomes especially in LMIC settings is still poorly understood. Applying the Shiffman and Smith (20) model to characterise prioritisation of zoonoses in India, our findings demonstrate, *inter alia*, that the complex and compartmentalised regulatory regime for zoonoses creates a policy milieu where political, socio-cultural and media influences can dominate prioritisation and management decisions.

Our findings highlight a challenging paradox. Several national and state-level interviewees acknowledged some drawbacks of the existing prioritisation process, including the sectorally-driven approach and the over-emphasis on exotic diseases (e.g., Zika, Nipah shaped by media coverage) at the expense of high burden of silent ones (e.g., KFD, leptospirosis, scrub typhus). At the same time, the same informants equally recognised that uncertainties of data (occasioned by the dearth of scientific evidence on certain focal zoonoses), lack of sensitive/point of care diagnostics hindering reporting, and the fact that different zoonoses impact populations differently renders the prioritisation of silent zoonoses (which are often under-reported, under-diagnosed, affecting populations that are marginal from health systems/economies and low severity) difficult. Synonymous with observation in the wider literature (25, 30), our findings suggest that media coverage was a critical driver of the extent of political visibility of certain focal zoonoses as this is often seen (in the face of limited scientific evidence) as tangible evidence of disease impact warranting action. Although the media coverage on a candidate disease was suggestive of public concern, there is a risk of potentially skewed prioritisation outcomes as diseases affecting poorer segments of society often remain obscure in the media space, even though marginalised poorer populations are known to be disproportionately affected by zoonotic diseases (3, 9, 18). This conveys the understanding that so-called public concern or media attention may sometimes not actually reflect the actual disease experiences and impacts warranting high political visibility (25, 30). It therefore follows that targeted training for media practitioners/journalist in risk communication and media engagement for disease managers alike is critical to help streamline and/or balance media narratives on disease outbreaks.

With respect to the characteristics of individual disease problems, the disease severity exemplified by mortality and case fatality significantly determined political prioritisation. The lack of standardised indices for measurement of severity given that different zoonoses affected populations differently and the unevenness in the evidence base for focal diseases renders collaborative-prioritisation (i.e., between animal and human health actors) difficult to achieve. For instance, whereas the seroprevalence of brucellosis is very high in animals, the corresponding human risk is low. Moreover, the lack of standard metrics in the animal health system to estimate disease burden (like the disability-adjusted life years (DALY) for humans) makes it difficult to estimate burden for chronic zoonotic diseases. The foregoing observation is also reflected in the contest of authority with respect to avenues for collaborative prioritisation, as animal health actors generally felt short-changed by the current state of play. Thus in facilitating collaborative-prioritisation, improvement in the ways burdens are captured in existing information systems across endemic as well as exotic diseases cannot be overemphasised. In furtherance to this, the Integrated health Information Portal (IHIP) is being developed (as a replacement for the existing IDSP) to afford an enhanced, collaborative and timely disease reporting platform. Besides, further understanding, investigating and developing indicators for the nature and extent of non-clinical, wider societal impact of zoonotic diseases, particularly for marginalised communities, rather than relying on mortality and morbidity metric only is paramount. In any event, the design and implementation of cross-sector action plans for priority diseases (e.g., the revised Action Plan for Avian Influenza) backed by strong political will at the highest decision-making strata remain critical in generating the requisite financial and non-financial incentives to foster and sustain collaboration of different actors in zoonotic disease management (9). Despite the leadership contestation between animal and human health actors cross-sectoral actors always navigate the policy spaces to collaborate in practice, sometimes leveraging inter-personal relationships where necessary. This is congruent with the notion of the dynamism of actors in circumventing identified barriers that constrained formal opportunities for cross-sectoral engagement for zoonoses management (9, 15, 31). It therefore follows that disease prioritisation is as political as it is technical.

### 4.1. Limitations

This study is not without limitations. We acknowledge that the use of a snowball sampling approach in the recruitment of key-informants could have potentially skewed the representation of interviewees in favour of a particular sub-group of interest in this study, noting that actors affiliated with the human health sector constituted the majority of our study sample. Nevertheless, our prior stakeholder analysis (9) and purposeful participant recruitment across a range of different sectors ensure that we were able to capture the population heterogeneity and represent the diverse perspectives on the topic. That said, the focus of the interviews on actors involved in zoonoses management at the national and Karnataka state only (due to time, resource constraints and the COVID-19 pandemic) might have shaped/influenced stakeholder priorities and perspectives, necessitating further examination overtime. Given that the majority of key-informants (*n* = 17) were interviewed after the COVID-19 pandemic could have raised their awareness about zoonoses, and therefore potentially ‘skewed’ their observations. In any event, India’s federal character implies a need for caution in the generalisability of the findings to other states of India due to the inherent political and culturally and economic diversity that exist in the 29 Indian states and the fact that health is a state subject, with disease prioritisation operationalised at the state level.

## 5. Conclusion

Looking ahead, the COVID-19 and One Health platforms could present a window of opportunity to galvanise political support for zoonoses. Proponents argue that One Health partnerships could alleviate information asymmetries and create opportunities for knowledge exchange on candidate zoonoses critical for effective prioritisation (31, 32). In addition, ongoing high-level conversations of replicating state-driven One Health models (e.g., Tamil Nadu) with state government endorsement could provide new convening mechanisms for leadership on zoonoses management. Moreover, strategically managing external frames, particularly on ‘silent’ neglected zoonotic diseases, could hold sway in convincing decision-makers that they should be concerned. A case in point is KFD, where the engagement of technical experts and disease managers helped in the external re-framing of the disease from a localised problem to a trans-state problem (warranting joined-up cross-sector efforts and evidence) elevating the disease profile on the state and national policy radar (3, 33). This suggests that building and sustaining strong science-policy interfaces are critical for bridging knowledge gaps on disease burdens and multi-factorial impacts for effective prioritisation and intervention.

## Data availability statement

The datasets presented in this article are not readily available because of sensitive participant information. Request to access the datasets should be directed to UK Centre for Ecology and Hydrology Institutional Data Access contact via FA (email: fesasa@ceh.ac.uk).

## Ethics statement

The studies involving humans were approved by the Research Ethics Committees of the Ashoka Trust for Research in Ecology and the Environment (IRB/CBC/0003/ATV/07/2018), Institute of Public Health (IPH) Bangalore (IEC-FR/04/2017) in India, and the The Liverpool School of Tropical Medicine Research Ethics Committee (favourable ethical opinion – reference numbers 17-062 and 20-051) in the United Kingdom. The studies were conducted in accordance with the local legislation and institutional requirements. The participants provided their written informed consent to participate in this study.

## Author contributions

FA developed the conceptual focus, reviewed the literature, synthesised its contents, analysed data, and wrote the manuscript. AS contributed to data gathering and curation, data analysis, and reviewed the manuscript. JY, SH, and BP contributed to the conceptual focus, data interpretation, and reviewed the manuscript. MC contributed to the data interpretation and reviewed the manuscript. All authors contributed to the article and approved the submitted version.

## Funding

The IndiaZooSystems project that led to these results is supported by the Medical Research Council (MRC), the Foreign, Commonwealth and Development Office (FCDO), the Economic and Social Research Council (ESRC) and Wellcome under the Health Systems Research Initiative (grant numbers MR/S012893/1 & MR/S012893/2) awarded to BP, MC, SH, and JY. Additional support was provided to FA and BP from the UK Research and Innovation Global Challenges Research Fund funded IndiaZooRisk project (grant number MR/T029846/1). The funders had no role in the study design, data collection and analysis, decision to publish, or preparation of the manuscript.

## Conflict of interest

The authors declare that they have no known competing financial interests or personal relationships that could have appeared to influence the study reported.

## Publisher’s note

All claims expressed in this article are solely those of the authors and do not necessarily represent those of their affiliated organizations, or those of the publisher, the editors and the reviewers. Any product that may be evaluated in this article, or claim that may be made by its manufacturer, is not guaranteed or endorsed by the publisher.

## Supplementary material

Supplementary Table 1 Interview Guide for Key-Informants.

Supplementary figure 1 Coding framework.
