# Supplementary material for: ‘It doesn’t happen how you think, it is very complex!’ Reconciling stakeholder priorities, evidence, and processes for zoonoses prioritisation in India
Source: Front Public Health. 2023 Aug 22;11:1228950. doi: 10.3389/fpubh.2023.1228950 (PMC10477356; doi:10.3389/fpubh.2023.1228950)
Supplement: Supplementary Table 1 — Interview Guide for Key-Informants. [file Table_1.DOCX]

**‘It doesn’t happen how you think, it is very complex!’ Reconciling stakeholder priorities, evidence and processes for zoonoses prioritisation in India**

**Interview Guide for Key Informants**

Thank you for your time for this interview. We have a few questions about the prioritisation of zoonotic diseases for intervention and research in India. Within our project, we are aiming to understand better the linkages between surveillance, knowledge, research and models within the human health–animal health-environment sectors in order to improve existing information systems that support disease surveillance and interventions in the country (or your state). Given your work and experience, your insights in this regard will be extremely valuable.

**General Information about respondent**

1. Details about the person, their job title, education background, geographical area/scale over which they operate.
2. Could you tell us about your experience and role with regards to zoonotic disease management? [Probes: role and years of involvement, whether involved in policy making, surveillance, human or animal or environmental interventions]

**Prioritisation of zoonoses in policy and knowledge gaps**

1. How and by which processes are zoonotic diseases prioritised in India by different cross-sectoral actors and at different geographic scales and regions? [Probe: Endemic, epidemic and exotic diseases]
2. Is there a formal prioritisation for zoonotic diseases in India?
3. If so, could you indicate whether you are in agreement with this prioritisation? i.e. is the current prioritisation justified? If not, could you explain why?
4. Who carries out the prioritisation? [Probe: extent of consideration of cross-sectoral components in the prioritisation process – what sectors are involved in the prioritisation?] Are there any sectors currently missing in this prioritisation process? Which ones? How could/should they be better involved in prioritisation?
5. If so, what factors informed the prioritisation?
6. To what extent is prioritisation linked to global burdens and distribution, mortality and morbidity rates, type of clinical or livelihood impacts, type of transmission, affected community (socio-economic status, urban-rural, gender, age)?
7. In your opinion, what are the case fatality rate, mortality rate or overall burdens in humans that matters for disease prioritisation and decision-making? [Probe: India burden/ case fatality] Why do you say this? (Probe to give examples)
8. Are pathogens given different treatment or weighting in terms of resources, interventions and research based on their status in the prioritisation? If so, please explain this. [Probe: differences in weighting for endemic, epidemic and exotic diseases]
9. How often is the prioritisation refreshed?
10. In your opinion, how is disease prioritisation affected by evidence gaps about pathogens? What is missing? [Probe: Disease burdens, CFR, economic and societal impacts, underreporting of endemic diseases etc.]
11. Are there opportunities to alter/ improve the way diseases are prioritised to reduce total burdens and address health inequalities? If so, please explain.

**Information and spatial decision support tools used inform zoonoses management**

1. In terms of zoonotic disease surveillance and interventions, what would you say are the major challenges that you face?
2. In terms of zoonotic disease surveillance and interventions, what are the main pieces of information that you need to guide your management?

Do you already use quantitative information, maps, forecasts or information systems to inform your management or work related to zoonotic diseases?

If so, can you describe

Which such information sources you use?

How you use them in your work?

Can you describe the extent to which you use the following common sources of information to inform ZD management? *Have a Likert or frequency scale e.g. every week or month, every quarter, once a year, never or rarely.*

Frequency scale: *1 (Never), 2 (Rarely), 3 (Sometimes/ once a year), 4 (often/ every quarter), 5 (Always/ every week or month)*

IDSP

IHIP

NADRES

Other (Please specify – up to 2 or 3 other sources)

1. How could models and information systems be improved to support zoonotic disease surveillance and interventions? Why do you say that?
2. To what extent do you think that linking information across sectors, for example within models or decision support tools, could improve management of zoonotic diseases? Why do you say this?
3. If you think this would be valuable, which sectors should be involved in the development of cross-sector decision support tools?
4. What would you say the key requirements of developing and integrating such cross-sector decision support tools into existing information systems?
5. Based on your experience do you have any suggestions to improve cross-sectoral collaboration towards effective zoonotic disease surveillance and interventions?
