# Supplementary material for: ‘It doesn’t happen how you think, it is very complex!’ Reconciling stakeholder priorities, evidence, and processes for zoonoses prioritisation in India
Source: Front Public Health. 2023 Aug 22;11:1228950. doi: 10.3389/fpubh.2023.1228950 (PMC10477356; doi:10.3389/fpubh.2023.1228950)
Supplement: Supplementary figure 1 — Coding framework. [file Presentation_1.PPTX]

## Slide 1
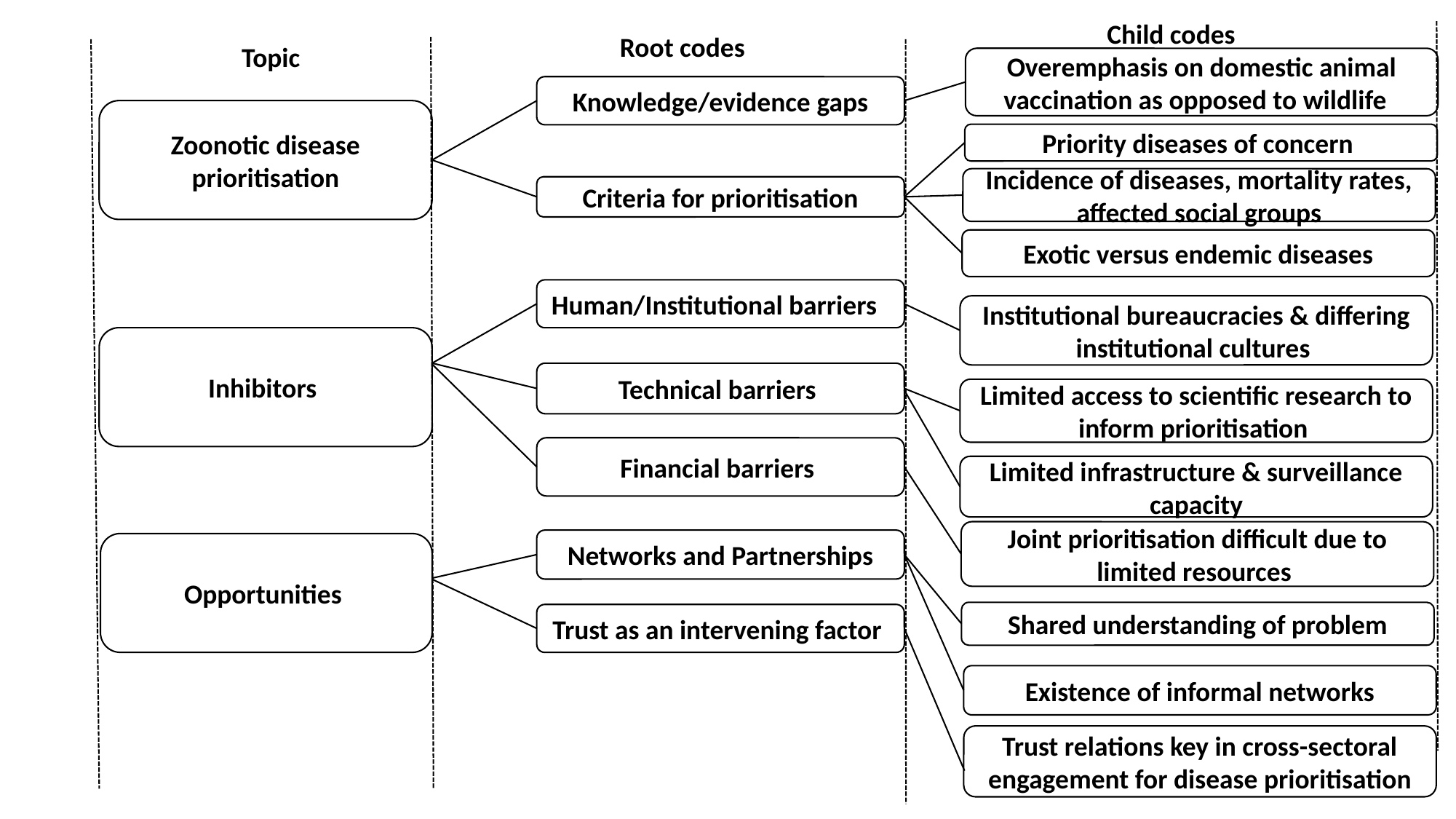

Child codes
Root codes
Topic
Overemphasis on domestic animal vaccination as opposed to wildlife
Knowledge/evidence gaps
Zoonotic disease prioritisation
Priority diseases of concern
Incidence of diseases, mortality rates, affected social groups
Criteria for prioritisation
Exotic versus endemic diseases
Human/Institutional barriers
Institutional bureaucracies & differing institutional cultures
Inhibitors
Technical barriers
Limited access to scientific research to inform prioritisation
Financial barriers
Limited infrastructure & surveillance capacity
Joint prioritisation difficult due to limited resources
Networks and Partnerships
Opportunities
Shared understanding of problem
Trust as an intervening factor
Existence of informal networks
Trust relations key in cross-sectoral engagement for disease prioritisation
